# The mode of action of plant associated *Burkholderia* against grey mould disease in grapevine revealed through traits and genomic analyses

**DOI:** 10.1038/s41598-020-76483-7

**Published:** 2020-11-10

**Authors:** Qassim Esmaeel, Cédric Jacquard, Lisa Sanchez, Christophe Clément, Essaid Ait Barka

**Affiliations:** Unité de Résistance Induite et Bioprotection des Plantes EA 4707, SFR Condorcet FR CNRS 3417, University of Reims-Champagne-Ardenne, Reims, France

**Keywords:** Microbiology, Plant sciences, Plant biotechnology

## Abstract

Plant-associated *Burkholderia* spp. have been shown to offer a promising alternative method that may address concerns with ecological issue associated with pesticide overuse in agriculture. However to date, little work has studied the role of *Burkholderia* species as biocontrol agents for grapevine pathogens. To this end, two *Burkholderia* strains, BE17 and BE24 isolated from the maize rhizosphere in France, were investigated to determine their biocontrol potential and their ability to induce systemic resistance against grey mould disease in grapevine. Results showed the capacity of both strains to inhibit spore germination and mycelium growth of *Botrytis cinerea*. Experimental inoculation with BE17 and BE24 showed a significant protection of bacterized-plantlets against grey mould compared to the non-bacterized control. BE17 and BE24-bacterized plants accumulated more reactive oxygen species and an increased callose deposition was observed in leaves of bacterized plantlets compared to the control plantlets. In bacterized plants, gene expression analysis subsequent to *B. cinerea* challenge showed that strains BE17 and BE24 significantly increased the relative transcript level of pathogenesis-related (PR) proteins *PR5* and *PR10*, two markers involved in the Salicylic acid (SA)-signaling pathway. Furthermore, in silico analysis of strains revealed the presence of genes involved in plant growth promotion and biocontrol highlighting the attractiveness of these strains for sustainable agricultural applications.

## Introduction

Grapevine (*Vitis vinifera* L.) is one of the major broadly cultivated fruit crops worldwide, and more particularly in France (https://www.oiv.int/). The economic importance of the plant is mainly related to its end-product, the wine. In spite of its relevance, the grapevine is exposed to different environmental stresses that significantly reduce its productivity. Among diseases affecting the grapevine, grey mould caused by *B. cinerea*, is one of the most threatening for sustainable grape growing^1^. This pathogen also affects the quality of both grapes and wine made from infected grapes. It is therefore responsible for significant economic losses in wine production^2^. Traditionally, chemically-based pesticides are mainly used to control the pathogen and related yield losses, but they are costly and have less efficiency towards a wide range of pathogens. On the other hand, chemical control does not provide a solution for sustainable agriculture as it has the potential to generate ecological problems such as the spread of pesticide resistance pathogens^3^. Therefore, opportunities to reduce the loss and enhance the productivity of the grapevine by using biological control strategies are of great interest.

Biological control including the use of plant growth promoting bacteria (PGPB) or their natural compounds has received remarkable attention as a promising method that will solve the ecological issue associated with pesticides overuse in the agriculture. These microorganisms represent a heterogeneous group of beneficial bacteria that naturally occur in the rhizosphere and form a significant relationship with the plant^4,5^. They reinforce plant resistance against pathogens and consequently reduce the significant loss of plant productivity through diverse mechanisms. The mechanisms include root colonization and competition with other microbes present in the environment, the production of antimicrobial peptides, and the induction of plant-mediated resistance responses^6–8^. After microbial perception through microbe-associated molecular patterns (PAMPs), plant cells evolve different strategies to switch on their defense mechanisms. The resistance depends strongly on the ability of PGPB to trigger the immune response known as PAMP-triggered immunity (PTI). The priming effects are characterized by different responses, including the accumulation of reactive oxygen species (ROS), callose deposition, and the expression of defense-related genes^9–12^. It has been shown that application of PGPB can enhance grapevine growth by increasing nutrients availability^13^ and inducing the systemic resistance toward biotic and abiotic stresses^14–17^.

The genus *Burkholderia* has been reported to be one of the most common soil bacteria. Different members of this genus have been isolated from different ecological niches including the rhizosphere, soils, plants, animals, and also humans^18–22^. It has been recently proposed to divide the genus *Burkholderia* into seven distinct groups; these include *Paraburkholderia, Robbsia, Pararobbsia, Mycetohabitans, Trinickia, Caballeronia*, and *Burkholderia* sensu stricto^22–26^. The latter includes species related to *B. mallei*, *B. pseudomallei*, *Burkholderia cepacia* complex (Bcc), and other pathogenic members^23,27^. Plant-associated species of *Paraburkholderia* and other members of Bcc group have been associated with biocontrol in a number of plants^28–32^. The application of these species in biocontrol is increasing and nowadays it constitutes promising candidates in agriculture not only as biocontrol, but also as biofertilizers^20,33–35^. Their beneficial effects are attributed to the production of secondary metabolites, volatile compounds and their ability to induce and/or increase the plant tolerance against different abiotic and biotic stresses^9,36^. However, to date, little work has studied the role of *Burkholderia* as a biocontrol agent for grapevine. To this end, two strains BE17 and BE24, isolated from the maize rhizosphere, were investigated for their potential biocontrol activity against the causal agent of grey mould disease. Furthermore, we have undertaken an investigation of the mechanism associated with the conferment of plant resistance to grey mould disease by BE17 and BE24. Genome sequence analysis of both strains revealed the presence of a set of genes involved in plant growth promotion and biocontrol abilities confirming their potential application as an alternative strategy for a sustainable agriculture.

## Results

### Identification and characterization of bacterial strains

**Figure 1:**
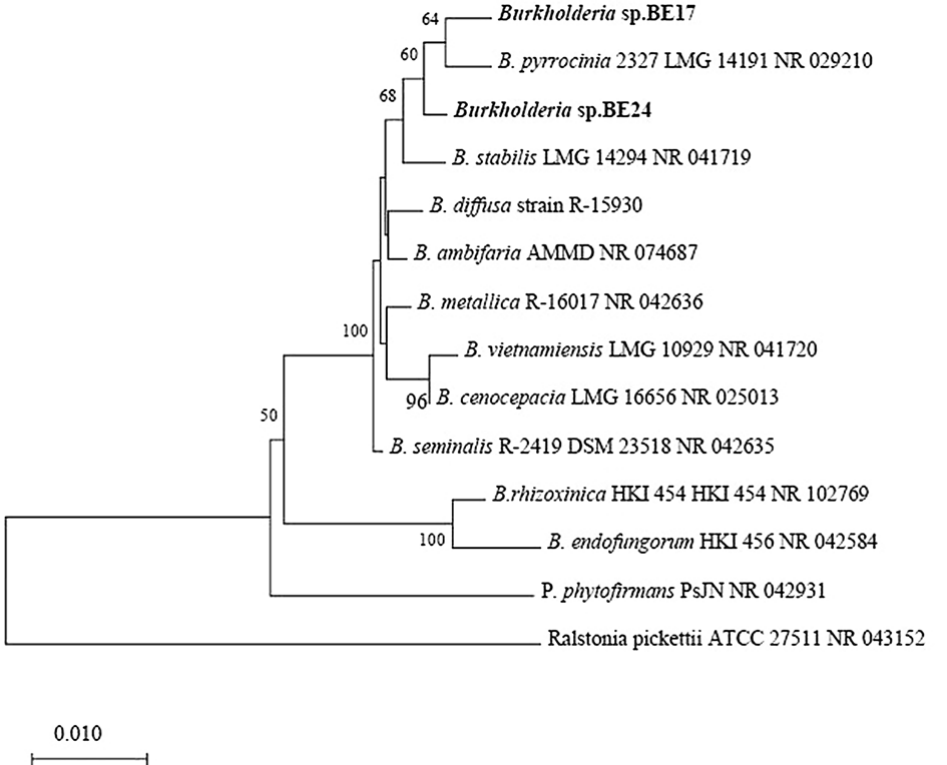
Phylogenetic tree of strains BE17 and BE24. The tree was constructed based on 16S rRNA gene sequences using MEGA version X with neighbor-joining method. Values at nodes indicate bootstrap values out of 1000 resampling. *Ralstonia pickettii* ATCC 27 was used as an outgroup. BE17 and BE24 are in bold.

The colonies of BE17 and BE24 were investigated for their morphological characteristics including shape, appearance, motility, and size. Strains BE17 and BE24 were grown on LB medium at 30 °C forming round and slightly raised colonies with 1.8–2.9 mm in diameter. They are Gram negative, rod shaped, and motile. They grew well on LB medium containing 0.5 and 1% NaCl and could grow at pH range 5 to 8. Supplementary Table S2 presents an overview of the results related to the different biochemical characteristics obtained with the BIOLOG GENIII microtiter plate (Hayward CA, USA). The 16S rRNA sequences of BE17 and BE24 shared similarity levels above 98% sequence identity with *B. ambifaria* AMMD, *B. metallica*, *B. seminalis*, and *B. cepacia*. Based on the 16S rRNA sequences, a phylogenetic tree was constructed using MEGA version X with neighbor-joining method which revealed that BE17 and BE24 belonged to genus *Burkholderia* (Fig. 1). In addition, to calculate the pair-wise average nucleotide identity (ANI) values with their closest known relatives, the draft genome sequences were submitted to the microbial genomes atlas (MiGA) webserver^37^. The analysis generated ANI values lower than 95% for strain BE24 and the closest relative was *B. cenocepacia* GCA 900446215 (94.27% ANI). For strain BE17, the closest relatives found were *B. pyrrocinia* NZ CP011503 (95.18% ANI) and *B. stabilis* NZ AP018111 (95.16% ANI). Moreover, digital DDH values of both strains were compared against all type strain genomes available in the TYGS database^38^. Results showed that strains BE17 and BE24 do not belong to any species found in TYGS database (DDH < 70%), indicating that strains BE17 and BE24 represent novel species of *Burkholderia*, belonging to the Bcc group. Therefore, the following names *Burkholderia* sp. BE17 and *Burkholderia* sp. BE24 are proposed. The ANI value between strains BE17 and BE24 was 91.91%, and the DDH value was less than 70% (52.4%), clearly indicating the genetic distinctiveness of strains BE17 and BE24.

### In vitro effect of strains BE17 and BE24 on mycelium growth and spore germination of *B. cinerea*

**Figure 2:**
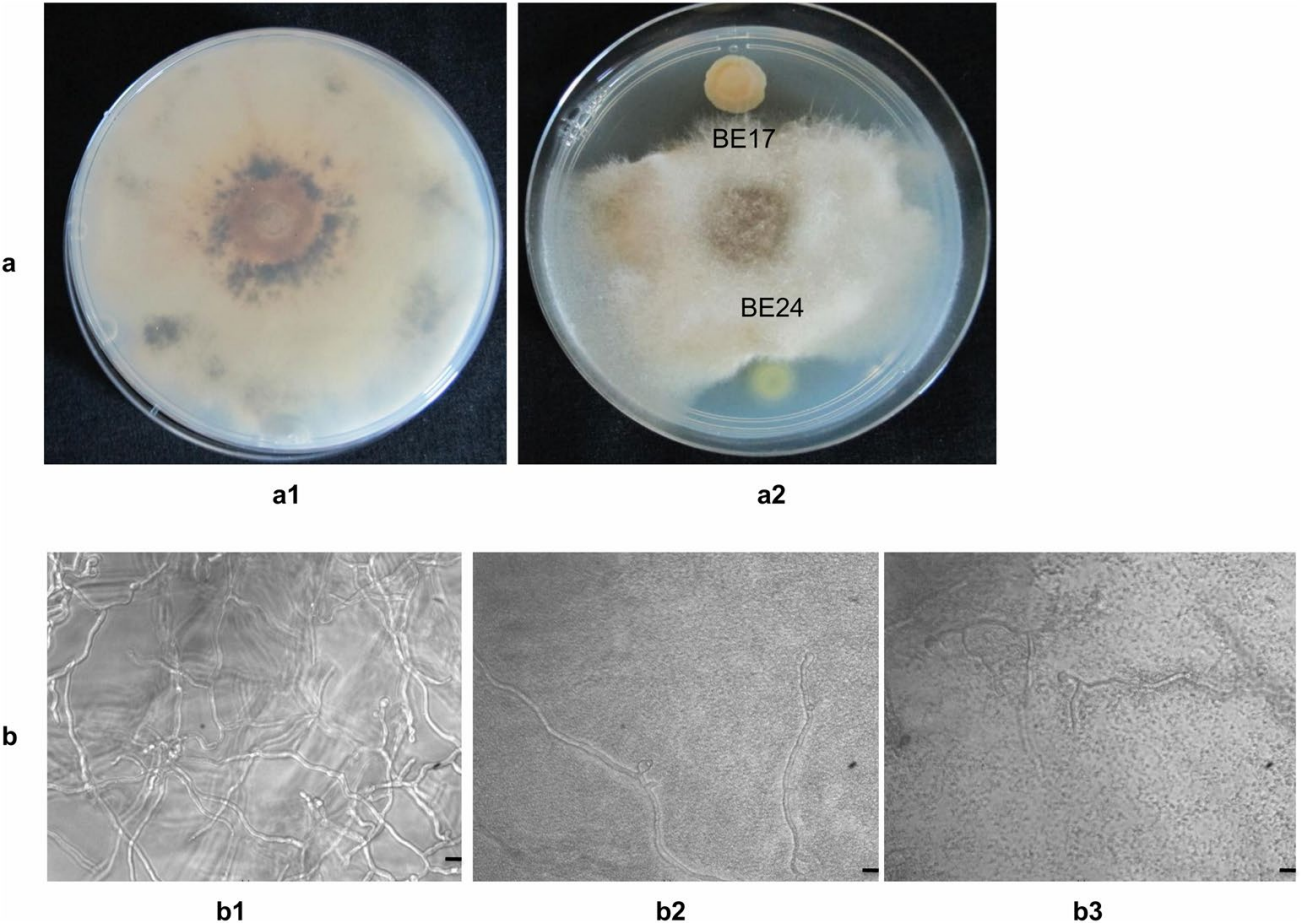
In vitro effect of strains BE17 and BE24 on *B. cinerea*. (**a)** *in vitro* fungal antagonism assay. (**a1)** *B. cinerea*. (**a2**) direct effect of BE17 and BE24 on *Botrytis*. (**b**) effect of BE17 and BE24 on spore germination of *B. cinerea.* (**b1**) *B. cinerea* germinated spores, (**b2,b3**) *B. cinerea* conidia were mixed with 10^6^ CFU/mL of BE17 and BE24, respectively. Bars = 100 µm.

Both strains were tested for their ability to inhibit spore germination and mycelium growth of *B. cinerea*. Results in Fig. 2a showed that strains BE17 and BE24 were able to inhibit the growth rate of *B. cinerea* in comparison with their respective controls. Strains BE17 and BE24 inhibited the spore germination and mycelium growth of *B. cinerea* in dual culture. Spore germination inhibition by strain BE24 was slightly greater than of the strain BE17. Spore germination of the pathogen was reduced by more than 90% when mixed with 10^6^ CFU/mL of the bacterial suspension (Fig. 2b). The finding suggested that both strains exhibit antifungal activity against *Botrytis*.

### Strains BE17 and BE24 protect grapevine against grey mould disease

**Figure 3:**
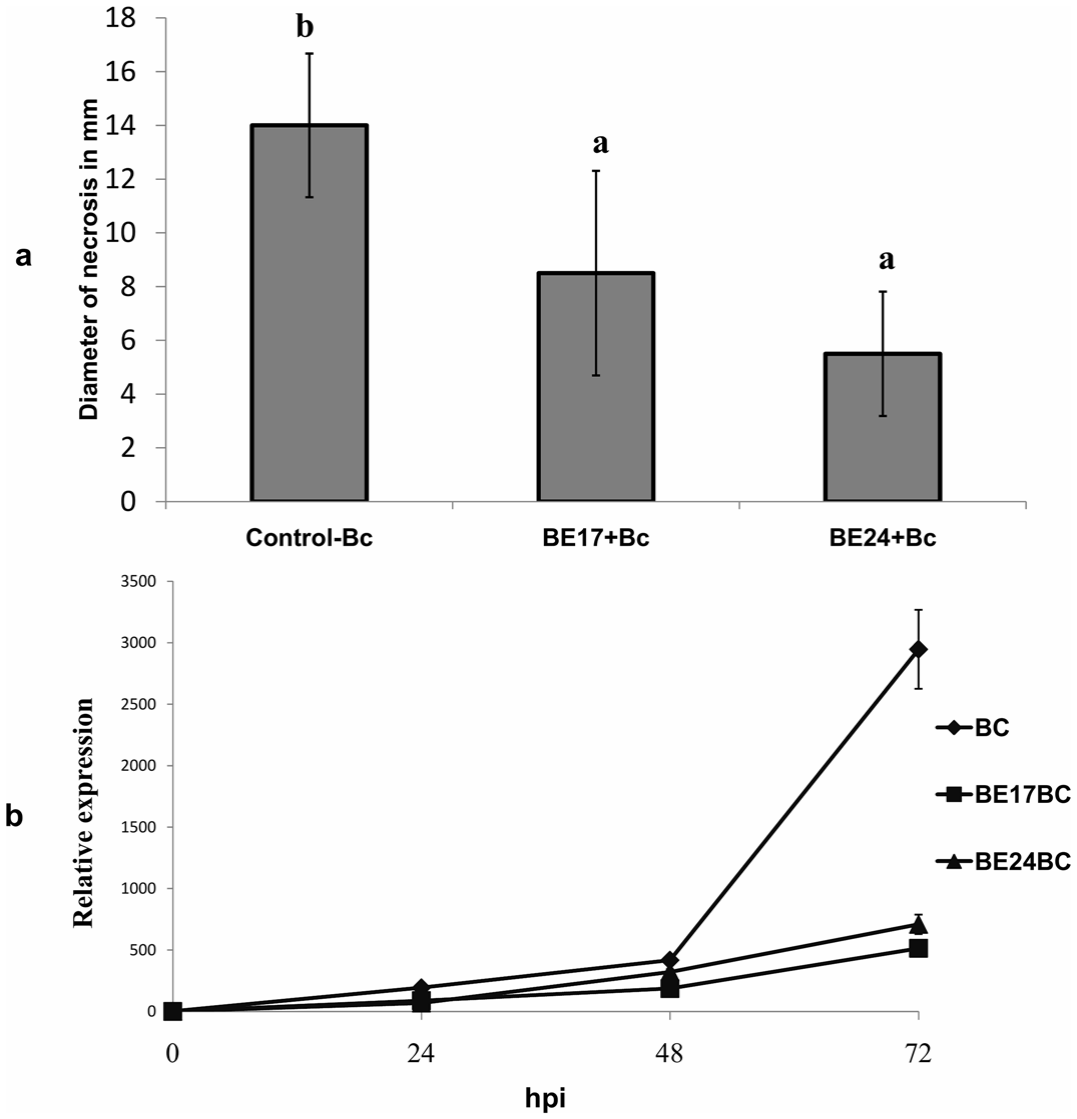
**(a)** *B. cinerea* bioassay on detached leaves inoculated or not with BE17 and BE24, 72 hpi. Control-Bc leaves of grapevine plantlets inoculated with PBS, BE17 + Bc and BE24 + Bc leaves of grapevine in vitro-plantlets inoculated with strain BE17 or BE24 and infected by *Botrytis*. Different letters indicate significant differences (P ≤ 0.05) between treatment and the non-treated control. **(b)** Relative expression analysis of *B. cinerea actin* by real time PCR in leaves of inoculated or non-inoculated plantlets with BE17 and BE24.

To further investigate the biocontrol properties of strains and their performance to defend the grapevine against grey mould, we evaluated infection on detached leaves with *Botrytis* on non-treated control vs. bacterized plantlets. Disease symptoms were evaluated by measuring the size of *Botrytis*-related necrosis. As shown in Fig. 3a, *Botrytis*-related necrosis was significantly reduced (p < 0.05) in detached leaves of bacterized plantlets treated with BE17 and BE24 compared to non-treated ones. The non-bacterized control resulted in more disease symptoms and the necrosis length attained 14 mm (Fig. 3a). The necrotic spot diameter was reduced by 57% and 36%; for BE24 and BE17, respectively. Furthermore, in order to quantify the growth rate of the fungus in treated *vs*. non-treated plantlets, in vitro-plantlets were sprayed with *B. cinerea* inoculum and then incubated at 25 °C in the growth chamber with a 16- and 8-h photoperiod. The expression level of the *B. cinerea actin* gene was quantified by qRT-PCR at 0, 24, 48, and 72 h post infection with the pathogen. As shown in Fig. 3b, the *Bc-Actin* transcript level in BE17 and BE24-treated plantlets was significantly lower than their respective control. Obviously, these results demonstrated that strain BE17 and BE24 remarkably reduced grey mould disease severity in the grapevine. Moreover, the growth development of the fungus in BE17 and BE24-bacterized plantlets was also visualized under a 3D microscope. As presented in Supplementary Figure S1, the fungal mycelium was significantly reduced at 48 h and 72 hpi in BE17 and BE24-bacterized plantlets compared to the non-treated control.

### In the presence of the pathogen, BE17 and BE24 primed ROS accumulation and callose deposition

**Figure 4:**
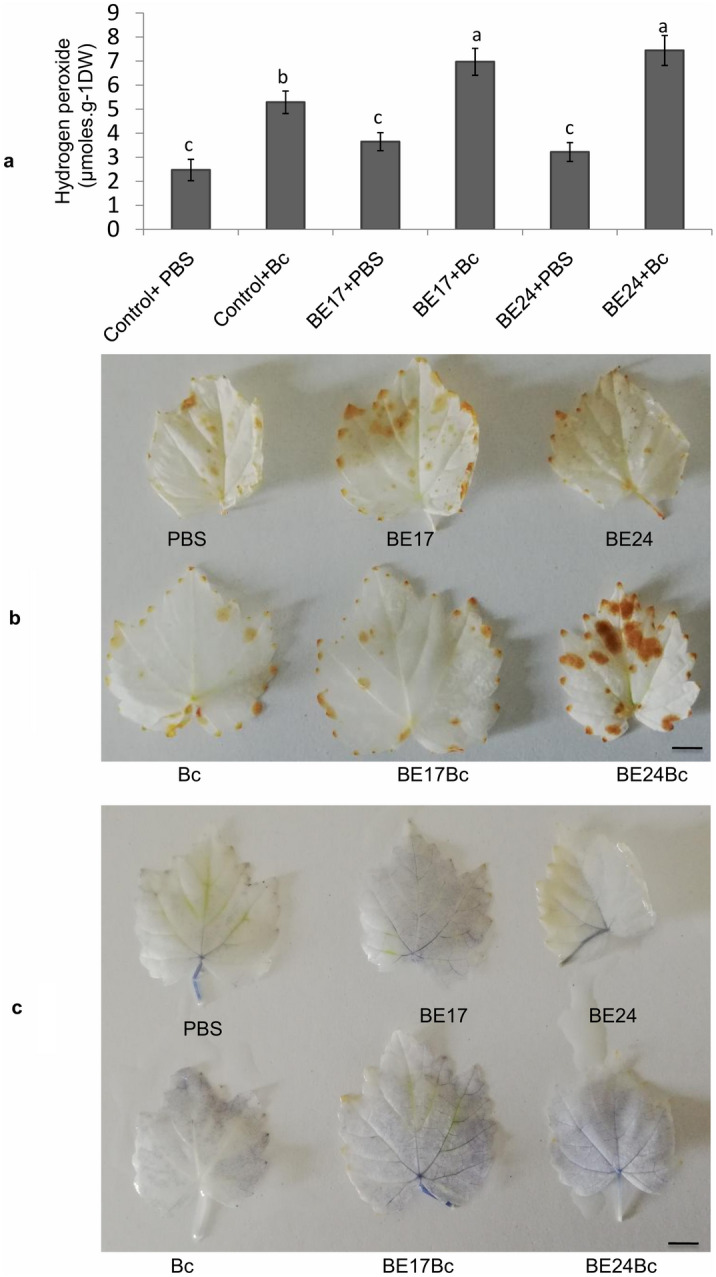
Accumulation of ROS in control and bacterized-plantlets after challenge with *B. cinerea*. (**a**) H_2_O_2_ content in control and bacteria-treated grapevine plantlets at 8hpi. Different letters indicate significant differences (P ≤ 0.05). (**b,c**) the histochemical detection of H_2_O_2_ and O_2_^-^ in leaves of control and bacterized plantlets 8 hpi. PBS grapevine plantlets inoculated with PBS, Bc grapevine plantlets inoculated with PBS and infected by *Botrytis*, BE17 and BE24 plantlets inoculated with strain BE17 or BE24, Bc + BE17 and Bc + BE24 plants inoculated with strain BE17 or BE24 and infected by *Botrytis*. Values shown are means ± SD of three independent repetitions (each repetition was realized in triplicate). Bars = 5 mm.

**Figure 5:**
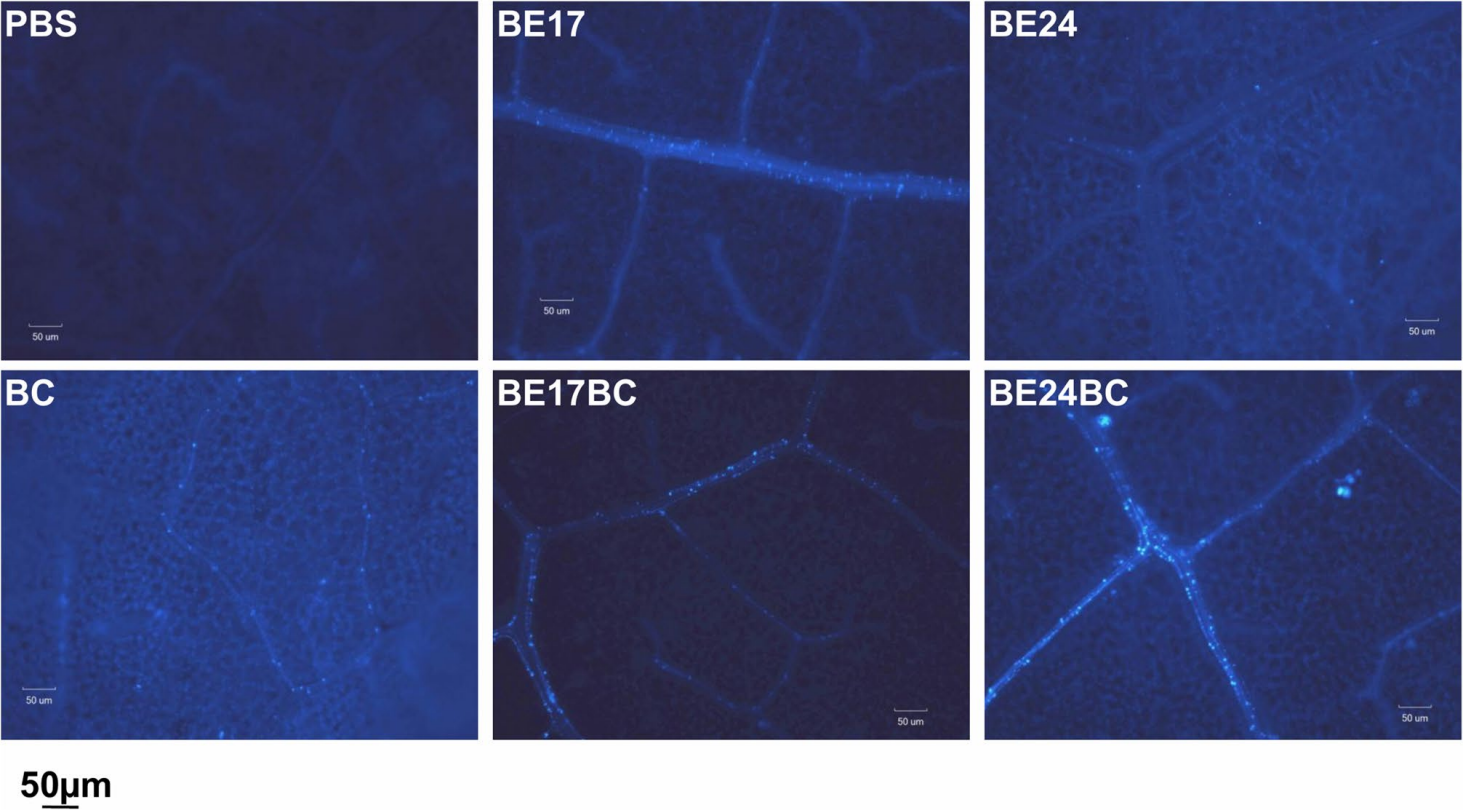
Callose deposition at 24 h after pathogen challenge (*B. cinerea*) in leaves of control and bacterized-plantlets. PBS plantlets inoculated with PBS, Bc plantlets inoculated with PBS and infected by *Botrytis*, BE17 and BE24 plantlets inoculated with strain BE17 or BE24, Bc + BE17 and Bc + BE24 plantlets inoculated with strain BE17 or BE24 and infected by *Botrytis*. Values shown are means ± SD of three independent repetitions (each repetition was realized in triplicate). Bars = 50 µm.

To further elucidate if strains BE17 and BE24 prime the basal level of reactive oxygen species (ROS) after being challenged with the pathogen, the histochemical detection of hydrogen peroxide (H_2_O_2_) and peroxidase (O_2_^-^) using 3, 3ʹ-diaminobenzidine (DAB) and nitrotetrazolium blue chloride (NBT) staining methods was evaluated at 8 h post infection (hpi) on control and bacterized leaves. Furthermore, the accumulation of H_2_O_2_ in control and bacteria-treated grapevine plantlets at 8 hpi was also investigated. Data shown in Fig. 4a indicated that the H_2_O_2_ level was 1.5 times primed in BE24- treated plantlets compared to non-treated ones. For BE17, the H_2_O_2_ content was 6.97 μmol g^−1^ DW. However, no significant differences were noted between treatment with BE17 and BE24. Interestingly, the production of H_2_O_2_ was not significantly observed in grapevine leaves in response to BE17 and BE24 inoculation. Moreover, H_2_O_2_and O_2_^-^were detected as a reddish-brown coloration and a dark blue stain, respectively, in leaves 8 h after the pathogen challenge in bacterized plantlets. Spots in control leaves were less intensified than bacterized leaves. In leaves of BE24-bacterized plantlets, colored dots were more noticeable than in leaves of BE17-treated plantlets (Fig. 4b,c). These results indicate the polymerization of DAB stain and the precipitation of blue formazan in the presence of H_2_O_2_ and O_2_^−^, respectively. For callose depositions, detached leaves from control and BE17 and BE24-treated plantlets were visualized under a fluorescence microscope using a DAPI filter 24 h post challenge with *B. cinerea.* As shown in Fig. 5, microscopic observation showed that subsequent to pathogen challenge, leaves of BE24 and BE17-treated plantlets delivered much more callose than those of the non-treated control. More callose deposition was observed in leaves of BE24-bacterized plantlets after infection with the pathogen. This result suggests that the inoculation of grapevine plantlets with BE17 or BE24 primed the callose deposition after the infection with the pathogen.

### BE17 and BE24 modulate different defense markers after pathogen infection

**Figure 6:**
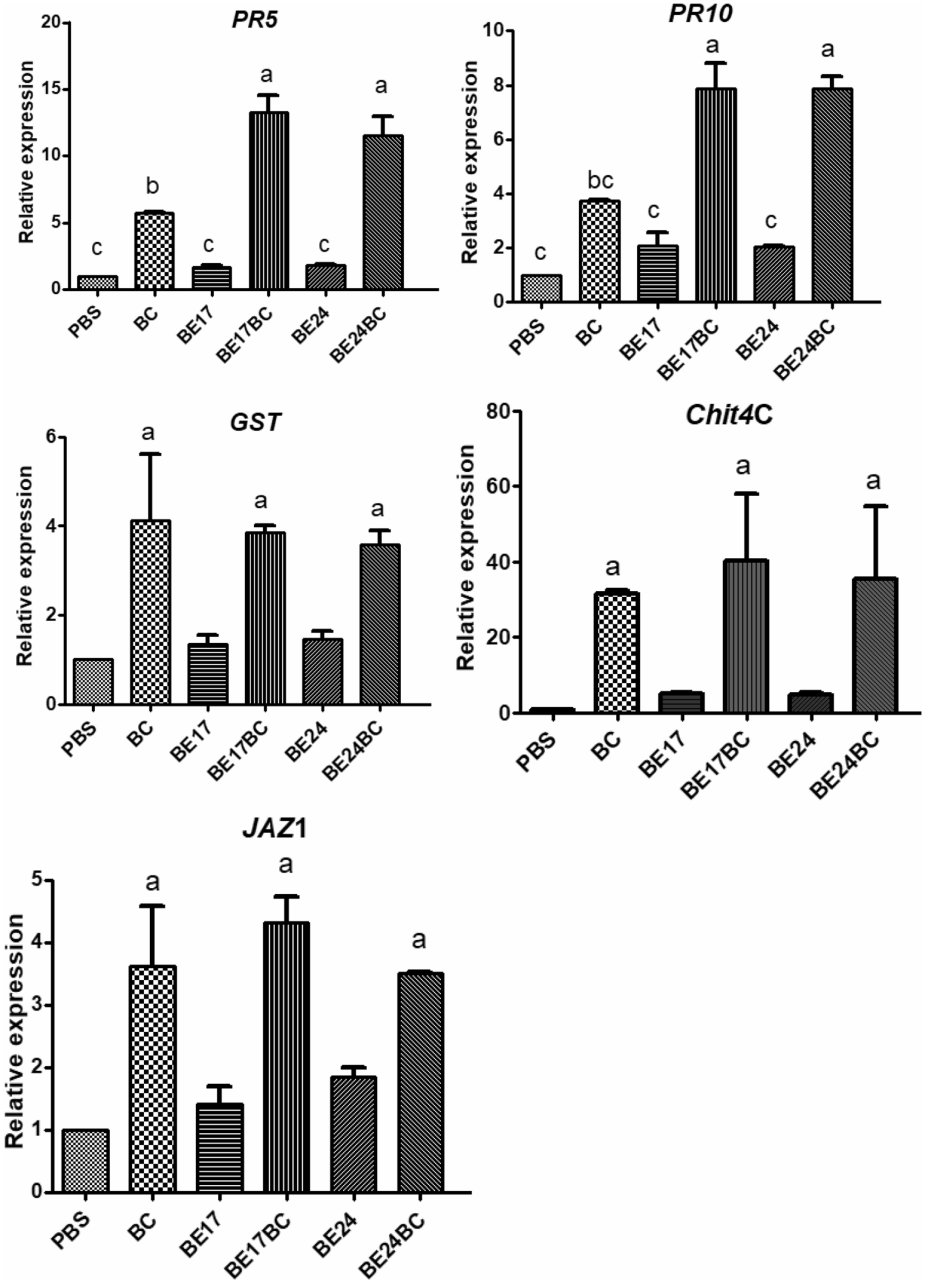
The expression of several defense markers in control and bacterized plantlets at 24 h post infection with *Botrytis*. Transcript accumulation of defense-related-genes, including pathogenesis related genes *PR5, PR10*, class IV chitinase (*Chit4C*), jasmonate zim-domain (*JAZ*) and glutathione S-transferase (*GST*) was carried out by qRT-PCR 24 h post challenge with the pathogen. The expression levels were normalized using two housekeeping genes [elongation factor 1-α (*EF1a*) and 60S ribosomal protein L18 (*60RSP*)] as internal controls. Results are expressed as the fold increase in transcript level compared to non-bacterized plantlets treated with PBS. PBS grapevine plantlets inoculated with PBS, Bc grapevine plantlets inoculated with PBS and infected by *Botrytis*, BE17 and BE24 grapevine plantlets inoculated with strain BE17 or BE24, Bc + BE17 and Bc + BE24 grapevine plantlets inoculated with strain BE17 or BE24 and infected by *Botrytis*. Values shown are means ± SD of three independent repetitions (each repetition was realized in triplicate). Different letters indicate significant differences (P ≤ 0.05) between treatment and the control-*Botrytis.*

The expression of several defense markers in control and bacterized plantlets at 24 h post infection with *Botrytis* was monitored. These markers include genes involved in the salicylic acid (SA)-signaling pathway; such as glutathione-*S*-transferase (*GST1*), thaumatin-like protein (*PR5*), and pathogenesis-related protein 10 (*PR10*); class IV chitinase (*Chit4*c), jasmonate zim-domain (*JAZ1*) as markers for jasmonic acid (JA)-signaling pathway. In bacterized plantlets, there was a slight modulation in the expression of defense genes markers; however, in non-bacterized plantlets, a significant expression was observed after the pathogen challenge (Fig. 6). *PR5* and *PR10* were up-regulated in BE17 and BE24-bacterized plantlets after pathogen infection. There were no significant differences between treatment with BE17 and BE24. The expression of *GST*, *Chit4C*, and *JAZ1* were not significantly modulated in both BE17 and BE24-treated plantlets as compared to non-bacterized plantlets.

### The complete genome analysis of BE12 and BE24

To provide insight into the genomic details of strains that confer antagonistic activities against grey mould disease, the whole genome sequencing of BE17 and BE24 was conducted. The genomes of strain BE17 and BE24 were sequenced at 30 ×, and 105 × coverage and assembled in 134 and 127 contigs, respectively. The entire genomes of BE17 and BE24 were 8,457,567 bp and 7,422,622 bp with GC content of 65.95% and 66.90%, respectively. In the complete genome of BE17 and BE24, a total of 8034 and 7090 genes were predicted, including 7958 and 7001 coding sequences (CDSs), 11 and 19 rRNA genes, 4 ncRNAs genes for each one, and 61 and 66 tRNA genes were predicted using microbial genome annotation and analysis platform (MicroScope) and NCBI (Table 1). Among the 7958 and 7001 coding genes predicted, 7524 (94%) and 6655 (94%) were protein coding genes, while 434 and 346 were non-protein coding genes for BE17 and BE24, respectively.

**Table 1:** Annotated genome features of *Burkholderia* isolates BE17 and BE24.

Features annotated	BE17	BE24
Genbank accession	WHNU00000000	WHNT00000000
Size (bp)	8,457,567	7,422,622
G + C content (%)	65.95	66.90
Genes (total)	8034	7090
Coding genes	7958	7001
Protein-coding genes	7524	6655
Non-coding protein	434	346
rRNA	11	19
tRNA	61	66
ncRNAs	4	4

**Figure 7:**
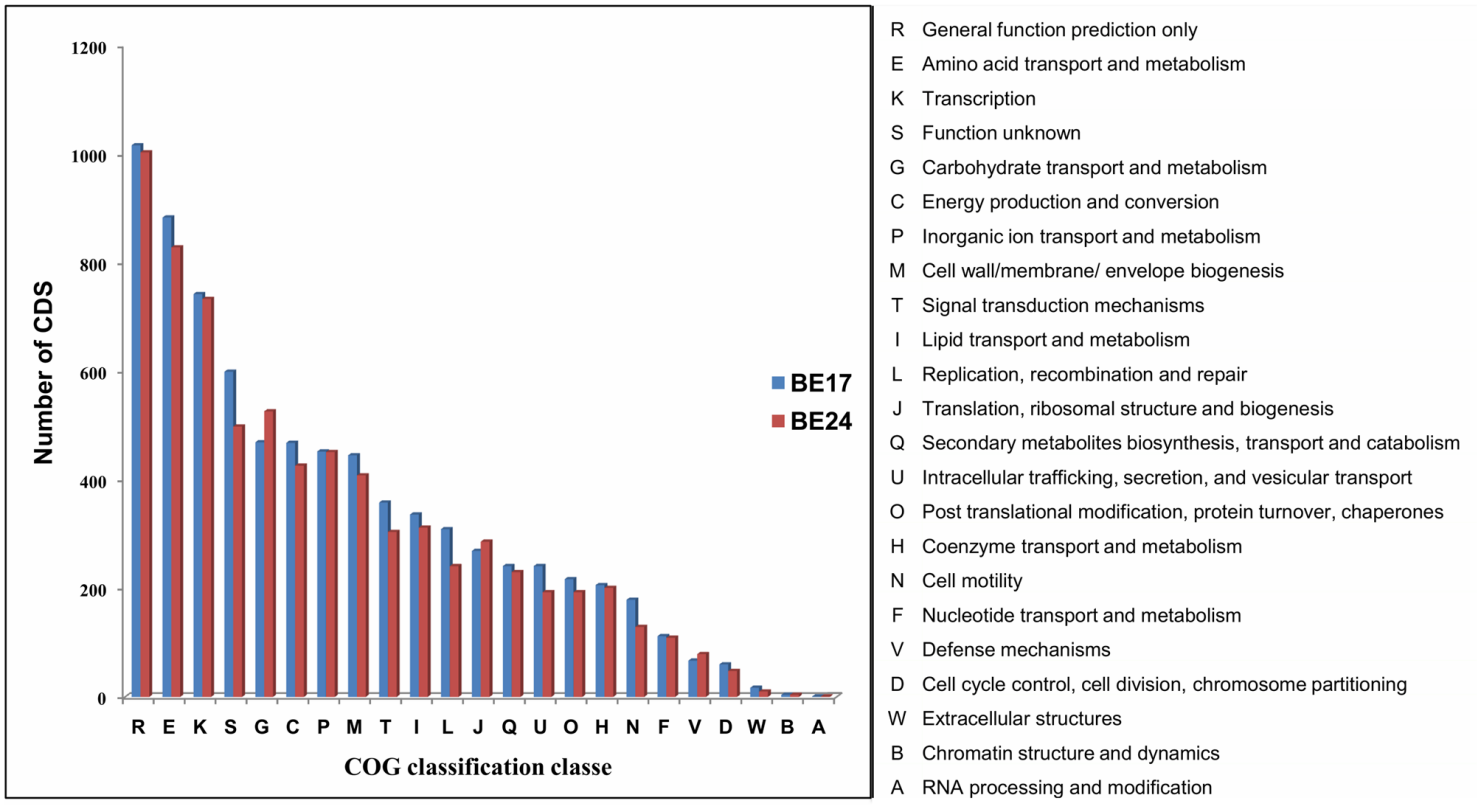
Clusters of orthologous groups (COGs) annotation of BE17 and BE24 predicted based on microbial genome annotation and analysis platform (MicroScope). The capital letters on the horizontal axis represent the function classes which are presented on the right of the graph and data on the vertical axis illustrate the number of CDS.

**Figure 8:**
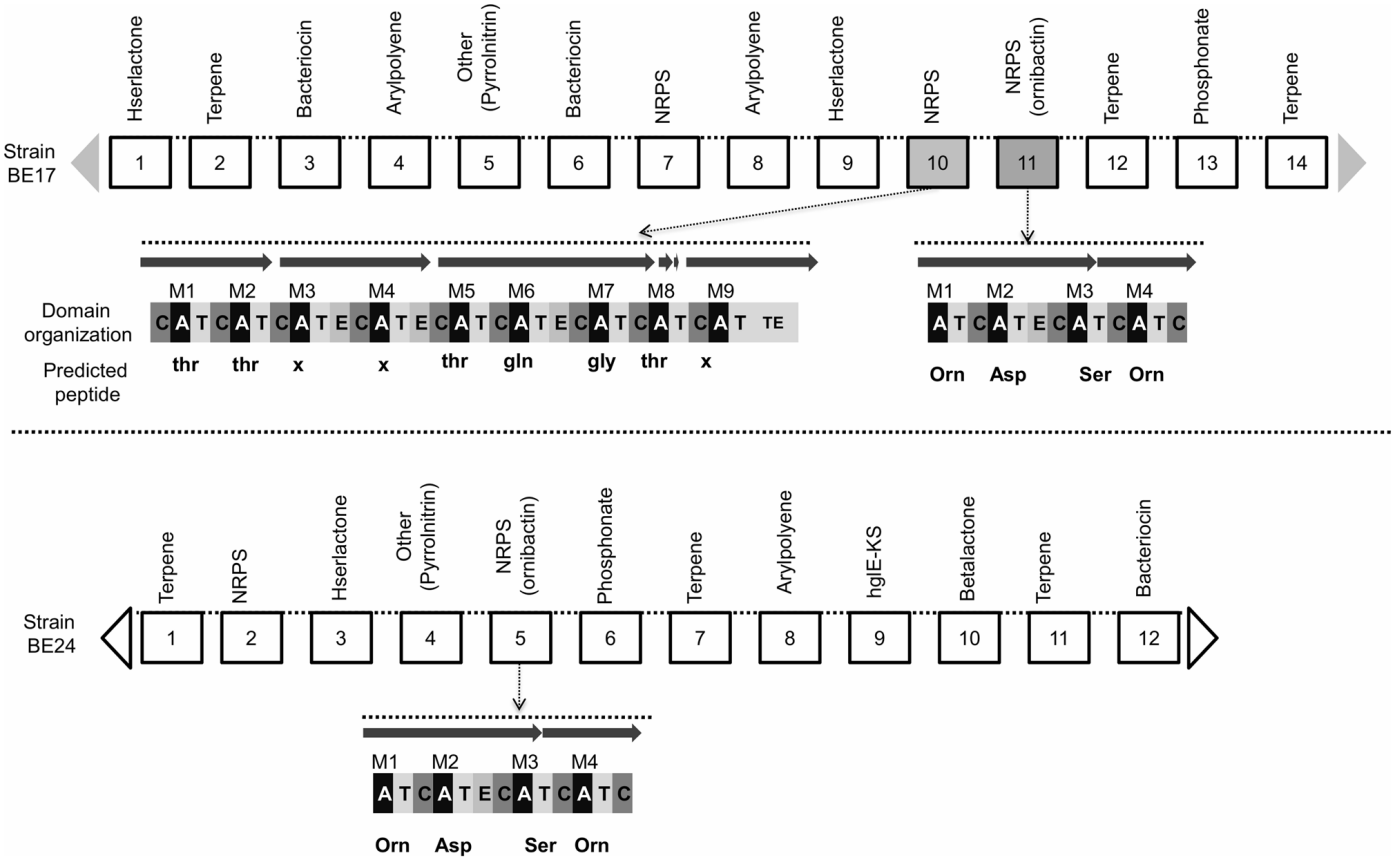
Potential of the predicted secondary metabolite biosynthesis gene clusters found in BE17 and BE24 genomes. Structure of NRPS gene clusters are presented in details, they are organized in modules which subdivided into domains. Domains abbreviations are as follow: Adenylation (A), condensation (C), thiolation (T), Epimerization (E), thioesterase (TE). Predicted monomers are highlighted under each A domain. X indicates that no significant prediction was obtained.

The putative function of the protein coding genes was predicted based on MicroScope platform and the clusters of orthologous groups (COGs) were assigned for the predicted genes. Amino acid transport and metabolism (E), transcription (K), carbohydrate transport and metabolism (G), and energy production and conversion (C) were among the most dominant classes (Fig. 7). Furthermore, to identify the essential gene clusters involved in the antagonism mechanism, we performed in silico analysis using the anti-SMASH server^39^. The exploration of BE17 and BE24 draft genomes pointed out the presence of numerous putative secondary metabolites gene clusters, including non-ribosomal peptide synthetase (NRPS), bacteriocin, arylpolyene, Hserlactone, terpene, phosphonate, and pyrrolnitrin (Fig. 8). Strains BE17 and BE24 both possess a cluster organized in a group of genes involved in ornibactin biosynthesis. The cluster includes two NRPs biosynthetic genes, and other genes coding for transporter, decorating and regulatory proteins (Fig. 8). The domains organization of the NRPSs and the substrate specificity of adenylation domains shared 100% similarity with the ornibactin synthesis. Furthermore, in the genome of BE17, we have identified another gene cluster consisting of four NRPS genes encoding nine NRPS modules. The synthetase is predicted to produce a peptide with nine monomers (Fig. 8). Furthermore, in silico analysis predicted the presence of genes involved in plant–microbe interactions (Supplementary Table S3). Among them, genes associated with plant growth promoting traits, including 1-aminocyclopropane-1-carboxylate (ACC) deaminase, genes involved in indole acetic acid (IAA) production. Besides, strains BE17 and BE24 possess several genes implicated in phosphate solubilization; these include the phosphoenolpyruvate carboxylase (Pepc) and the PQQ dependent glucose dehydrogenase (GDH) which play a crucial role in the biosynthesis of organic acids involved in P-solubilization. Genes involved in phosphate metabolism including the ABC transporter complex (*pst*A, *pst*B, *pst*C, and *pst*S), and the Pho regulon were also found in both genomes. In addition, the positive effect of BE17 and BE24 on the grapevine is apparently helped by the presence of a set of genes associated with signaling, motility, chemotaxis, detoxification, and quorum sensing (Supplementary Table S3). In our analysis of BE17 and BE24 draft genomes, respectively, we have found 74 and 48 genes implicated in the biosynthesis of flagella formation, 41 and 59 genes involved in signaling, and others related to cell host adhesion and root colonization. Moreover, BE17 and BE24 both possess genes coding for putative cell wall hydrolytic enzymes allowing the entry of beneficial bacteria into the host cell to control grey mould disease by direct inhibition (Supplementary Table S3). In addition, during our analysis, different types of secretion systems including T2SS, T3SS, T4SS, and T6SS were founded in both strains.

## Discussion

The present study demonstrated the capacity of *Burkholderia* strains BE17 and BE24 to control grey mould disease caused by *B. cinerea* in grapevine by direct and ISR effects. Phylogenetic analysis using 16S rRNA sequences revealed that BE17 and BE24 are closely related to *B. ambifaria* AMMD, *B. metallica*, *B. seminalis*, and *B. cepacia*, all of which were previously reported to play an important role in biological control strategies in many plant species, including tomato, pea, sweet corn, citrus, cucumber and soybean^9,30–32^. Members of *Burkholderia* genus are well known to occupy a wide range of ecological niches^21^. They are phenotypically diverse and are assembled into seven branching genera including *Paraburkholderia*, *Robbsia*, *Pararobbsia*, *Mycetohabitans*, *Trinickia*, *Caballeronia*, and *Burkholderia* sensu stricto^22–26^. The highest diversity of this genus is believed to be related to the large genome encoding different secondary metabolites and extracellular enzymes, which enable them to be more competitive and robust in extreme environments^40^. Isolates BE17 and BE24 belong to the *B. cepacia* complex (Bcc) clade; a group of plant, animals and human pathogens as well as plant growth promoting species^23,27,41,42^. Different members mediate high beneficial interactions and are commercially exploited as biopesticides^27^. However, the major problem of this group is associated with their abilities to cause opportunistic infections, especially those with cystic fibrosis. Therefore, the main question is to understand *Burkholderia* as being both beneficial and pathogenic bacteria. From the discovery of *Burkholderia* until present, the evolutionary study of *Burkholderia* continues to demonstrate their diversity and genomics, which further spurred our interest to use these bacteria as beneficial biopesticides. The ability of different Bcc strains to suppress or kill fungal pathogens was a key feature which brought the initial exploitation of *Burkholderia* for agricultural applications. In in vitro assays, strains BE17 and BE24 inhibited the mycelium growth and germination rate of *Botrytis.* The antifungal activity could be due to the production of hydrolytic enzymes and secondary metabolites by bacterial strains^43^. Another mechanism is that bacterial strains depleted nutrients in the medium, thereby affecting the growth of the pathogen^8^. It has been reported that the antifungal effects of *Burkholderia* species appear to result from the production of metabolites as pyrrolnitrin, burkholdines, siderophore, xylocandin, and cepacidin^44–48^. Moreover, in a recent study of Mullins et al. (2019), different strains of *B. ambifaria* were shown to produce the antifungal agent called cepacin, which directly mediate crop protection against pathogens^49^.

Developing the biodiversity of the biological control system through screening for new strains is a mean of achieving a good level to control a wide range of plant pathogens. During our analysis, our core aim was to screen for new *Burkholderia* strains with the dual ability to colonize the grapevine and also provide biocontrol effect against pathogens. In this study, to potentially apply these isolates for grapevine protection program, we highlighted the promising biocontrol activity of two isolates towards *B. cinerea*, the causal agent of grey mould disease^1^. Based on results of the in vivo assay, strains BE17 and BE24 were effective and significantly reduced grey mould disease severity. Members of plant-associated *Burkholderia* have been reported as biostimulants or biocontrol agents acting as phytopathogen antagonists in the environment inside or outside the host plant^32,42^. Different studies have demonstrated that the biocontrol activity of *Burkholderia* is linked to their ability to produced antibiotics, siderophores, and other metabolites^41,46^. However, little work has studied the mechanism underlying the positive effect associated with *Burkholderia*. These can be either directly, by interfering with the pathogen through antibiosis, or indirectly, by priming plants protective response or inducing plant resistance^50,51^. In the present study, we also explored the ability of BE17 and BE24 to induce a systemic resistance response and activate grapevine defense against *B. cinerea*. As reported, different strains of *Burkholderia* can trigger the activation of systemic resistance in the plant^9,51^. Bacteria-mediated priming is described by different physiological responses, including changes in reactive oxygen species (ROS) level, callose deposition, and activation of plant defense related genes^52^. In grapevine, the accumulation of ROS including H_2_O_2_ is considered to be signaling compounds involved in the activation of defense responses^10^. BE17 and BE24 bacterized plantlets accumulated more reactive oxygen species (ROS) in leaves than control 8 h post infection. Moreover, previous studies highlighted the significant role of callose synthesis in plant defense responses^11^. In this study, more callose deposition was observed in leaves of BE17- and BE24-bacterized plantlets confirming the role of callose deposition in grapevine defense responses against pathogen attacks^51,53^.

In bacterized plantlets, genes expression analysis after *Botrytis* challenge showed that strains BE17 and BE24 significantly increased the relative transcript level of pathogenesis-related (PR) proteins *PR5* and *PR10*, two markers involved in the SA-signaling pathway^54^. Other studies reported that some PGPR induce systemic resistance through the activation of the SA-signaling pathway^55^. Our results concluded that strains BE17 and BE24 can trigger the grapevine immunity through an activation of the SA-signaling pathway resulting in a systemic resistance response against grey mould disease under control conditions.

Genome sequence is a good tool allowing the prediction of all genes that are relevant for the beneficial interaction between plants and bacteria. To provide further information dedicated to the beneficial effects associated with strains, we sequenced the whole genomes of BE17 and BE24 followed by coding sequence annotation. Accordingly, we predicted the presence of a set of genes involved in plant growth-promoting and biocontrol properties. In both strains, we detected some genes that are associated with motility and chemotaxis which seem to be required for efficient plant colonization^56^. Furthermore, the genome analysis of strains BE17 and BE17 predicted the presence of genes encoding for cell wall degrading enzymes, which allow the entrance into the host, resulting in a better colonization and, consequently help strains to exert their biocontrol activities directly inside the host’s tissues^56,57^. As reported in previous studies, the presence of genes involved in PGPR properties can contribute to better access to limited nutrients^5,58^. In our analysis, some PGPR-associated genes were detected, including genes responsible for siderophore biosynthesis, phosphate solubilization and IAA production, and bacterial secretion systems. Indeed, the presence of these genes allow strains to efficiently compete for nutrients with other pathogens, which in turn increase their PGPR and biocontrol activities^57^. In addition to PGPR traits, strains BE17 and BE24 harbour many secondary metabolite biosynthetic gene clusters (BGCs) that are involved in the production of metabolites including NRPS that can indirectly improve the plant growth by suppressing phytopathogens^41,59^. Identification of all these genes supports the biocontrol and PGP capabilities of the strains. Thus it can be concluded that strains BE17 and BE24 offer different mechanisms of action towards pathogens, confirming their potential for biocontrol applications.

## Conclusions

The present study demonstrated the capacity of *Burkholderia* strains to control grey mould disease caused by *B. cinerea* in grapevine by direct and ISR effects. Both strains significantly inhibited mycelium development and spore germination of *B. cinerea* and were able to induce ISR in grapevine against grey mould disease. Further, analysis of strain BE17 and BE24 genomes revealed the presence of a set of genes involved in PGP, including siderophore, phosphate solubilization, IAA production and other genes that are involved in antibiosis confirming their abilities as biocontrol agents against phytopathogens. Thus it can be concluded that strains BE17 and BE24 offer different mechanisms of action towards *B. cinerea*, confirming their biocontrol proprieties and potential exploitation towards sustainable agriculture.

## Materials and methods

### Bacterial isolation

Strains BE17 and BE24 were isolated in 2016 from maize rhizosphere cultivated in the northeast of France. 10 g of each rhizospheric soil sample was suspended in 90 mL of sterile NaCl solution and shaken at 240 rpm for 10 min. The mixture was serially diluted after which plates with Luria Bertani (LB) medium (tryptone 10 g/L; yeast extract 5 g/L; NaCl 5 g/L; pH 7.2) were inoculated with 0.1 mL of suspensions from the appropriated dilution. Isolated colonies were purified by subsequent streaking at 30 °C onto fresh plates on LB. Two strains, named as BE17 and BE24 were found to exhibit an antagonistic effect against *B. cinerea*. Strains were maintained in LB broth with 20% glycerol at − 80 °C for long term storage.

### Culture conditions and inoculum preparation

Isolated strains of *Burkholderia* were grown on LB medium at 30 °C. For plant bacterization experiments, the cultures were collected after centrifugation at 4500 *g* for 15 min, washed, and resuspended in phosphate-buffer saline (PBS) (10 mM of NaH2PO4, 2.7 mM of KCl, 1.8 mM of KH2PO4, 137 mM of NaCl, pH 6.8). The bacterial concentration was determined by pectrophotometry (OD_600_ nm) and adjusted with PBS (D_0_ = 0.8) to 10^8^ CFU/mL. *B. cinerea* (obtained from Dr. Y. Brygoo, INRA, Versailles, France) (Genbank accessions FQ790245 to FQ790362) was maintained on potato dextrose agar (PDA) (Sigma-Aldrich, MO, USA) at 25^◦^C, in the dark.

### Screening for antifungal activity (inhibition of spore germination and mycelium growth)

Spores of *B. cinerea* were harvested from 20-day old PDA plates by adding 5 to 10 mL of PDB medium and scratching the surface with a sterile L-shaped spreader. After that, the suspension was collected and then filtered to remove hyphae. A Malassez counting chamber was used to count conidial concentration. Strains BE17 and BE24 (10^6^ CFU/mL ) were prepared in 96-well microplates containing 100 µL of PDB medium and then the spore suspension was added to each well to a final concentration of 5,000 spores. The plates were then incubated for 24 h at 25 °C, in the dark. Germinated spores were observed using inverted light microscope. Experiments were conducted in triplicate and results were expressed in terms of the percentage of spores germinated relative to the control. For the vegetative growth assay, PDA plates were inoculated with *Burkholderia* strains and then 5-mm inverted plug of *B. cinerea* was placed on PDA plates. The plates were incubated at 25 °C for 5–7 days. Results were expressed by measuring diameter of mycelium growth inhibition zone compared to control plates.

### Molecular identification of strains BE17 and BE24

A single colony of each isolate was grown overnight at 30 °C in LB medium (5 mL); then, 1 mL of the bacterial suspension was centrifuged at 13,000–16,000 × *g* for 2 min to obtain the bacterial pellet. Total DNA was extracted using the Wizard Genomic Purification DNA Kit (Promega Corp., Madison, WI, USA) according to manufacturer instructions. DNA pellet was washed with 70% ethanol, dried and resuspended in 50 μL of DNase-free water and incubated overnight at 4 °C for solubilization. Isolates BE17 and BE24 were identified by sequencing the 16S rRNA gene using primers FD2 and RP1 (Supplementary Table S1) following the method described by Esmaeel et al. (2019)^50^. 16S rRNA sequences (> 1420 nt) obtained were submitted to the basic local alignment search tool (BLAST) provided online by the National Center for Biotechnology Information (NCBI, Bethesda, MD, USA). The phylogenetic tree was built using the neighbor-joining algorithms and kimura two-parameter model via MEGA version X^60^. The 16S rRNA gene sequences of strains BE17 and BE24 have been deposited in GenBank under accession numbers MT912593 and MT912594.

### Plant material

In vitro-plantlets of grapevine (*Vitis vinifera* L. cv. Chardonnay clone 7535) were regenerated by nodal explants on Murashige-Skoog (MS) medium as described by Ait Barka et al. (2006)^14^. Plantlets were grown at 25 °C in a growth chamber under white fluorescent light (200 µmol/m^2^ s), with a 16- and 8-h photoperiod.

### Bacterial treatment and pathogen infection

Strains BE17 and BE24 were investigated for their ability to reduce the incidence of grey mould in grapevine. Bacterial cultures of BE17 and BE24, grown overnight at 30 °C with continuous shaking (160 rpm), were collected in their exponential growth phase (18 h) by centrifugation at 4500 *g* for 15 min. Pellets were then washed and then re-suspended in a sterile PBS buffer. The final concentration was adjusted to 10^8^ CFU/mL. Two hundred microliter of the bacterial suspension was applied at the root level of each grapevine in vitro-plantlets. Control plantlets were treated with sterile PBS. One week later, control and bacterized plantlets were transferred into a magenta box containing 120 g of soil. The plantlets were then incubated for an additional 10 days before infection with *B. cinerea*. The *B. cinerea* inoculum prepared from 20-day-old PDA plates was adjusted to a concentration of 10^5^ conidia/mL using sterile PBS. Subsequently, grapevine in vitro-plantlets were sprayed with *B. cinerea* inoculum and then incubated at 25 °C in the growth chamber with a 16- and 8-h photoperiod. Control plantlets were sprayed with sterile PBS. For gene expression analysis, leaves were collected at 0, 24, 48, and 72 hpi. H_2_O_2_ production and callose deposition were also evaluated on infected and non-infected leaves 8 and 24 hpi, respectively. Detached leaves assay was used for pathogen infection and disease evaluation. Briefly, leaves of both control and bacterized plantlets were excised and placed on Petri dishes containing 0.5% of agar and then inoculated with 5 µL of *B. cinerea* suspension (10^5^ conidia/mL). Infected leaves were incubated in a growth chamber at 25 °C. The severity of grey mould disease was evaluated by measuring the necrosis diameter.

### In situ detection of H_2_O_2_ and O_2_^-^

Detection of H_2_O_2_ and O_2_^-^ was evaluated at 8 hpi on control and bacterized leaves from three independent experiments. For the histochemical detection of H_2_O_2_ and O_2_^-^ anion, DAB and NBT staining methods were used according to Kumar et al. (2014), with some modifications^61^. Briefly, fresh leaves were excised and immersed in 1 mg/mL of the 3,3-diaminobenzidine (DAB) or 1 mg/mL of nitroblue tetrazolium (NBT), and incubated at 37 °C for 6 h. Leaves were then destained in boiling ethanol (96%) for 10 min, following which images were captured on a plain white background under uniform lighting. Furthermore, the H_2_O_2_ content in control and bacteria-treated grapevine plantlets at 8 hpi was also measured according to the protocol previously described^51^. The H_2_O_2_ content was calculated relative to a standard curve. Data are the means ± standard deviations of three independent experiments realized in triplicate.

### Callose depositions

Callose depositions were visualized under a fluorescence microscope using a DAPI filter as previously described^62^. Detached leaves from control and bacterized plantlets were collected at 24 h post challenge with *B. cinerea*. Leaves were destained in ethanol 96% overnight and thereafter washed in 150 mM K_2_HPO_4_ for 30 min. After that, leaves were incubated in staining solution (0.01% aniline blue in 150 mM K_2_HPO_4_) during 2 h and then images were acquired by an epifluorescence microscope with UV filter (Bp, 340–380; LP, 425 nm). This experiment was repeated three times in triplicate.

### Microscopic observation of *B. cinerea* development

Development of *B. cinerea* was studied in leaves of control and bacterized plantlets at 24, 48, and 72 hpi with *B. cinerea* according to the protocol described previously^63^. Mycelium development was visualized under a 3D microscope (Keyence, France).

### Gene expression analysis

All primers used are listed in the Supplementary Table S1. For gene expression analysis, leaves collected from control and bacterized plantlets at 0, 24 h post infection (hpi) were grounded in liquid nitrogen. To quantify the growth level of *B. cinerea in planta*, the quantification of Bc*Actin* was evaluated at 0, 24, 48, and 72 h post infection with *B. cinerea*. 75 mg of leaf ground powder was used to extract total RNA using a PlantRNA purification reagent according to manufacturer instructions (Invitrogen, Pontoise, France). 150 ng of RNA was used for complementary DNA (cDNA) synthesis using the verso cDNA synthesis kit (Thermo Electron, Courteboeuf, France) according to the manufacturer. The expression of several defense markers from the different functional classes was analyzed by real-time quantitative PCR using the Chromo4 system (BIO-RAD, Hercules, CA, USA) and the SYBR Green Master Mix PCR kit (Applied Biosystems, Foster City, CA, USA) according to the protocol previously described by Miotto-Vilanova et al. (2016)^51^. The results presented correspond to the mean and standard deviation of duplicated reaction of three independent experiments.

### Genome analysis and annotation

Total DNA was extracted using the Wizard Genomic Purification DNA Kit (Promega Corp., Madison, WI, USA). Genomes were sequenced at MicrobesNG (https://www.microbesng.uk) using Illumina MiSeq and HiSeq 2500 technology platforms, with 2–250-bp paired-end reads. Reference genomes were defined using Kraken^64^. Raw reads were assembled by De novo assembly using SPAdes (https://bioinf.spbau.ru/spades). Assembled draft genome sequences were annotated using the microbial genome annotation and analysis platform (MicroScope)^65^. For in silico analysis and to predict secondary metabolites, the MicroScope platform was used. Whole Genome Shotgun projects of strains BE17 and BE24 have been deposited at GenBank under the accession numbers WHNU00000000 and WHNT00000000, respectively. Versions described in this paper are versions WHNU01000000 and WHNT01000000, respectively.

### Statistical analysis

Statistical analyses were performed using GraphPad Prism version 5.00 for Windows, GraphPad Software, San Diego California USA (https://www.graphpad.com). One-way ANOVA with Tukey test (α = 0.05) was used to calculate the significant differences between samples.

## Supplementary information

Supplementary Information
