# Supplementary material for: The mode of action of plant associated Burkholderia against grey mould disease in grapevine revealed through traits and genomic analyses
Source: Sci Rep. 2020 Nov 10;10:19393. doi: 10.1038/s41598-020-76483-7 (PMC7655954; doi:10.1038/s41598-020-76483-7)
Supplement: Supplementary file 1 — Supplementary Information [file 41598_2020_76483_MOESM1_ESM.pdf]

Scientific reports

Supplementary material

The mode of action of plant associated *Burkholderia* against grey mould disease in grapevine revealed through traits and genomic analyses

Qassim Esmaeel[1*], Cédric Jacquard[1], Lisa Sanchez[1], Christophe Clément[1], Essaid Ait Barka[1*]

[1]Unité de Résistance Induite et Bioprotection des Plantes EA 4707, SFR Condorcet FR CNRS 3417, University of Reims-Champagne-Ardenne, Reims, France

***Correspondence:** [Prof. Essaid Ait Barka and Dr. Qassim ESMAEEL, Unité de Résistance Induite et Bioprotection des Plantes EA 4707, SFR Condorcet FR CNRS 3417, University of Reims-Champagne-Ardenne, Reims, France]. **E-mail**: [ea.barka@univ-reims.fr & qassim.esmaeel@univ-reims.fr]

Supplementary Table S1. List of primers used in this study

Primers	Sequences **(5'-----> 3')**	References
FD2	AGAGTTTGATCATGGCTCAG	1
RP1	ACGGTTACCTTGTTACGACTT	
Vv60RSPF	ATCTACCTCAAGCTCCTAGTC	2
Vv60RSPR	CAATCTTGTCCTCCTTTCCT	
VvEF1aF	AACCAAAATATCCGGAGTAAAAGA	2
VvEF1aR	GAACTGGGTGCTTGATAGGC	
Chit4cF	TCGAATGCGATGGTGGAAA	3
Chit4cR	TCCCCTGTCGAAACACCAAG	
PR5F	TTTGATCATCCTTAGGGTAGCTGTAA	
PR5R	ACCAATGAGTACTGTTGCAATTCC	
PR10F	CGTTAAGGGCGGCAAAGAG	4
PR10R	GCATCAGGGTGTGCCAAGA	
GST1F	TGCATGGAGGAGGAGTTCGT	5
GST1R	CAAGGCTATATCCCCATTTTCTTC	
JAZ1F	GAGAAGGGCACGTTTGGAGA	6
JAZ1R	CATCGTCGTTGTTGTCGCTG	

References

1 Asghari, S., Harighi, B., Mozafari, A., Esmaeel, Q. & Ait Barka, E. Screening of endophytic bacteria isolated from domesticated and wild growing grapevines as potential biological control agents against crown gall disease. *BioControl* **64**, 723–735. doi:10.1007/s10526-019-09963-z (2019).

2 Sawicki, M. *et al.* Distinct regulation in inflorescence carbohydrate metabolism according to grapevine cultivars during floral development. *Physiol. Plant.* **154**, 447-467. doi:10.1111/ppl.12321 (2015).

3 Esmaeel, Q., Jacquard, C., Clément, C., Sanchez, L. & Ait Barka, E. Genome sequencing and traits analysis of *Burkholderia* strains reveal a promising biocontrol effect against grey mould disease in grapevine (*Vitis vinifera* L.). *World J. Microbiol. Biotechnol.* **35**. doi:10.1007/s11274-019-2613-1 (2019).

4 Castro, A. J. *et al.* The herbicide flumioxazin stimulates pathogenesis-related gene expression and enzyme activities in *Vitis vinifera*. *Physiol. Plant.* **134**, 453-463. doi:10.1111/j.1399-3054.2008.01151.x (2008).

5 Letousey, P. *et al.* Early events prior to visual symptoms in the apoplectic form of grapevine esca disease. *Phytopathol.* **100**, 424-431. doi:10.1094/phyto-100-5-0424 (2010).

6 Miotto-Vilanova, L. *et al. Burkholderia phytofirmans* PsJN confers grapevine resistance against *Botrytis cinerea* via a direct antimicrobial effect combined with a better resource mobilization. *Front. Plant Sci.* **7**, 1236. doi:10.3389/fpls.2016.01236 (2016).

Supplementary Table S2. Biochemical characteristics for BE17 AND BE24 based on BIOLOG GENIII microtiter plate (Hayward CA, USA) as recommended by manufacturers.

Oxidation of	BE17	BE24	Oxidation of	BE17	BE24	Oxidation of	BE17	BE24
Acetic acid	+	-	***β*** -hydroxy-D,L-butyric acid	+	+	D-serine	-	-
Acetoacetic acid	±	-	Inosine:	-	-	L-serine	+	+
L-alanine	+	+	α -ketobutyric acid	-	±	D-sorbitol:	+	+
Ɣ-aminobutyric acid	+	+	α -ketoglutaric acid	-	-	Stachyose	-	-
D-arabitol	+	+	L-lactic acid	+	+	Sucrose	+	+
L-arginine	+	+	D-lactic acid methylester	-	-	D-trehalose	-	-
D-aspartic acid	+	-	D-lactose	-	+	D-turanose	±	-
Bromo-succinic acid	+	±	D-malic acid	-	±	Tween 40	+	+
Citric acid	+	+	L-malic acid	+	+	**Growth in presence of:**		
Dextrin	±	±	D-maltose:	-	-	Aztreonam	+	+
D-fructose	+	+	D-mannitol	+	+	Fusidic acid	+	-
D-fructose-6-PO4	+	+	D-mannose:	±	+	Guanidine HCl	±	±
D-fucose	-	±	D-melibiose	-	±	Incomycin	+	+
L-fucose	+	+	3-methylglucose	+	±	Lithium chloride	-	-
Formic acid	+	+	***β*** -methyl-D-glucoside	-	-	Minocycline	+	-

Gelatin	+	-	Methylpyruvate	+	+	NaCl (1%)	+	+
Gentiobiose	-	±	Mucic acid	+	+	NaCl (4%)	-	-
L-galactonic acid lactone:	+	+	Myo-inositol	+	+	NaCl (8%)	-	-
D-galactose	+	+	N-acetyl-D-galactosamine	±	-	Nalidixic acid	±	+
D-galacturonic acid:	+	+	N-acetyl- ***β***-D-mannosamine	-	-	Niaproof 4	+	+
D-gluconic acid	+	+	N-acetyl neuraminic acid	-	-	pH 5	+	+
α-D-glucose:	+	+	Pectin	+	+	pH 6	+	+
D-glucose-6-PO4	+	+	P-hydroxyphenylacetic acid:	±	+	Potassium tellurite	+	+
Glucuronamide	+	+	Propionic acid:	±	+	Rifamycin SV	+	+
D-glucuronic acid	+	+	L-pyroglutamic acid	+	+	Sodium bromate	-	-
L-glutamic acid	+	+	Quinic acid	+	+	Sodium butyrate	-	±
Glycerol	+	+	D-raffinose:	±	-	1% sodium lactate	+	+
Glycyl-L-proline	±	-	L-rhamnose	-	±	Tetrazolium blue:	+	+
L-histidine	+	+	D-saccharic acid	+	+	Tetrazolium violet	+	+
α -hydroxybutyric acid	-	-	D-salicin	+	-	Troleandomycin	+	+
						Vancomycin	+	+

-: negative, +: positive, ±: borderline

Supplemental Table S3. Genomic screening of *Burkholderia* sp. BE17 and *Burkholderia* sp. BE24 for genes involved in the plant-bacteria relationship and functions possibly involved in biocontrol. Numbers indicate the nb of associated genes found, +: presence of associated genes, -: absent

Class or gene function		BE17	BE24
**I. Genes involved in plant growth promoting**			
IAA production	Indole acetamide hydrolase	2	2
	Indole-3-pyruvate	1	1
	Indole-3-glycerol phosphate synthase	1	1
ACC deamination	1-aminocyclopropane-1-carboxylate deaminase	1	1
Phosphate solubilization	Phosphoenolpyruvate carboxylase (Pepc)	+	+
	The PQQ dependent glucose dehydrogenase	+	+
	ABC transporter complex (pstA, pstB, pstC, and pstS)	+	+
	The Pho regulon (PhoB-PhoR, PhoU)	+	+
	Exopolyphosphatase (Ppx)	+	+
	Polyphosphate kinase (Ppk)	+	+
**II. Genes involved in plant colonization**			
Chemotaxis and swarming motility	Type IV pili	+	+
	Flagella	74 (+)	48 (+)
	Chemotaxis	+	+
	Methyl-accepting chemotaxis protein II	66	66
	Chemotaxis protein	29 (+)	28 (+)
Signaling	Two compenent system	32	39
	Sigma factors	9	20
Quorum-sensing	Acyl-homoserine-lactone based system	+	+
Plant cell wall degrading enzymes	Endoglucanase	+	+
	Polygalacturonase	+	+
	Glycoside hydrolyses	+	+
	Beta-glucosidase	+	+
	glycosidaes	+	+
	Alpha/beta hydrolase	+	+
Fe uptake	TonB-dependent receptor	14	22
	Siderophore biosynthesis	+	+
Secretion systems	T1SS	−	−
	T2SS	+	+
	T3SS	+	+
	T4SS	+	+
	T5SS	−	−
	T6SSa and b	+	+
Detoxification	Glutathione S-transferase	16	15
	Catalases	2	5

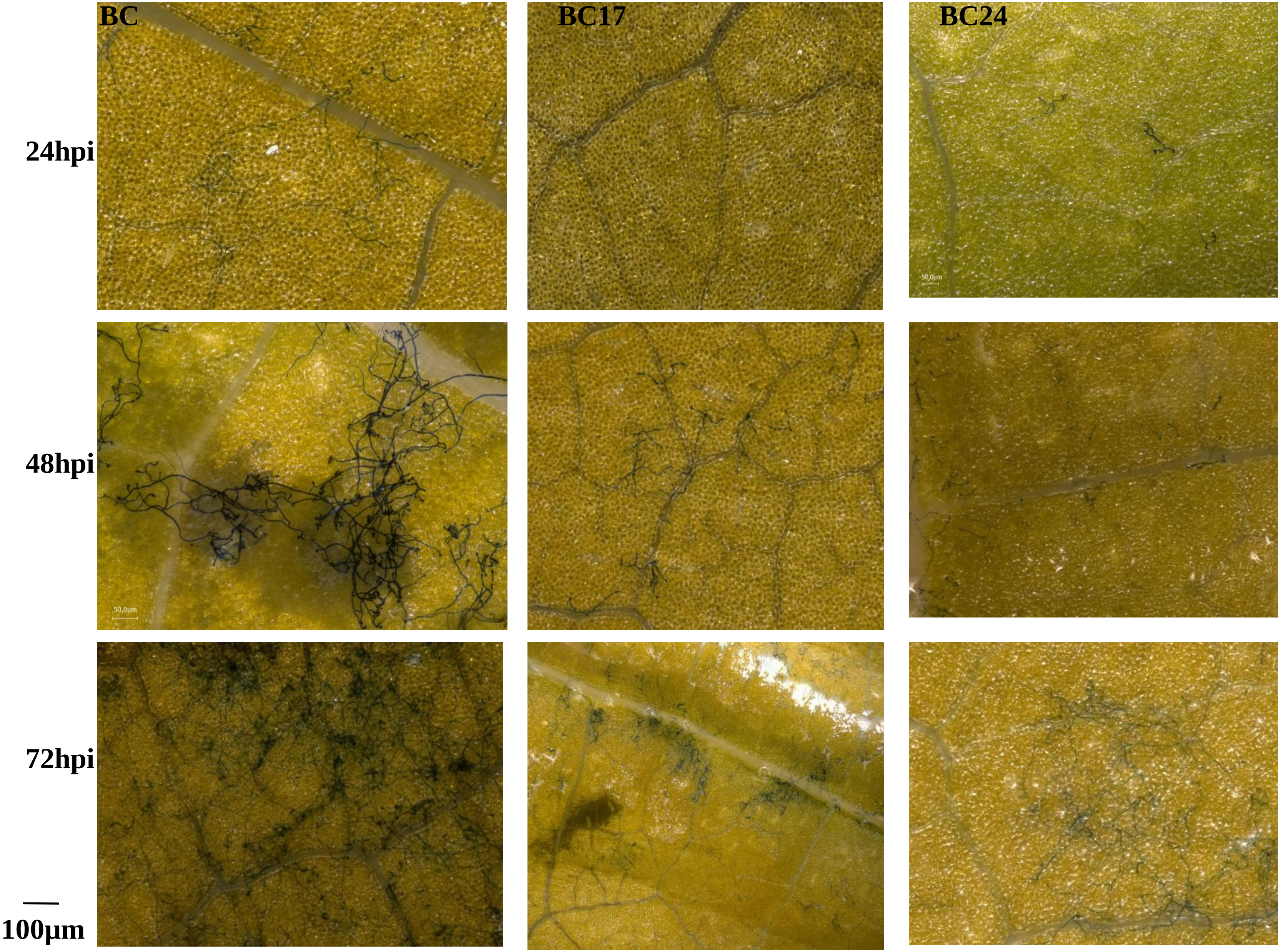

Supplementary Figure S1. Development of *B. cinerea* in plantlets inoculated or not with isolates BE17 and BE24
